# Recurrence distance analysis for clinical target volume optimization in glioblastoma

**DOI:** 10.1016/j.ctro.2026.101258

**Published:** 2026-08-12

**Authors:** Patrick L.Y. Tang, Marion Smits, Erik D. van Werkhoven, Remi A. Nout, Esther A.H. Warnert, Alejandra Méndez Romero

**Affiliations:** a Brain Tumor Center, Erasmus MC Cancer Institute, University Medical Center Rotterdam, Rotterdam, Netherlands; b Department of Radiology & Nuclear Medicine, Erasmus MC, University Medical Center Rotterdam, Rotterdam, Netherlands; c Department of Radiotherapy, Erasmus MC Cancer Institute, University Medical Center Rotterdam, Rotterdam, Netherlands; d Medical Delta, Delft, Netherlands

**Keywords:** Glioblastoma, Radiotherapy, Recurrence distance, Precision medicine, Pattern of failure, Clinical target volume

## Abstract

**Purpose:**

To analyze the pattern of failure in patients with glioblastoma through recurrence distance analysis, and to evaluate the feasibility of reduced and personalized clinical target volume (CTV-)margins.

**Methods:**

This study included 201 patients with IDH-wildtype glioblastoma who received standard chemoradiation between 2012 and 2022 and subsequently developed tumor recurrence. From these patients, clinical variables, radiotherapy treatment plans, and imaging data were collected. The recurrence distance was defined as the minimum isotropic expansion from the gross tumor volume (GTV) required to encompass 80% of the recurrence volume (RD_80_). Multiple linear regression modeling with backward elimination was performed to identify variables that correlate with the RD_80_.

**Results:**

Median time between the last radiotherapy fraction and progressive disease was 6.8 months (IQR: 4.1–11.8 months). The median RD_80_ was 1.2 mm (IQR: 0–6.0 mm). For 80% of the patients in our cohort, the RD_80_ was ≤10.4 mm. In the final multiple linear regression model, the log-transformed GTV volume (β = −0.70, *p* = 3.9e-7), involvement of the subventricular zone (β = 0.53, *p* = 0.019) and involvement of the subgranular zone (β = 0.51, *p* = 0.032) were significantly associated with the log-transformed RD_80_.

**Conclusion:**

This work highlights the potential of reduced CTV-margins in patients with glioblastoma, supporting further prospective evaluation.

## Highlights

• We analyzed glioblastoma recurrence distances in a large retrospective cohort.
• Eighty percent of the recurrences occur within 10 mm of the GTV.
• Smaller GTVs are associated with greater recurrence distances.
• Neurogenic zone involvement is associated with greater recurrence distances.

## 1 Introduction

Radiotherapy is one of the pillars in glioblastoma management. As glioblastoma is notorious for its extensive tumor infiltration, the 2023 ESTRO-EANO guidelines [1] recommend a 15 mm clinical target volume (CTV)-margin, adjusted for anatomical barriers, around the gross tumor volume (GTV). In the majority of cases, this expansion adequately encompasses tumor infiltration. However, these large target volumes can also encompass considerable amounts of healthy brain tissue, which increases the risk of radiotherapy-related adverse events [2], [3], [4], [5]. Identifying opportunities to safely reduce the CTV-margin, without compromising tumor control, may allow radiotherapy to achieve similar therapeutic efficacy while preserving the quality of life.

Several studies have highlighted the potential of various smaller CTV-margins [6], [7], [8], [9], showing a pattern of failure consistent with that of cohorts treated with a conventional CTV-margin. For instance, Paulsson et al. [8] retrospectively compared the pattern of failure in 161 patients treated with different CTV-margins and found no statistically significant differences in pattern of failure: In-field recurrence was observed in 79%, 77%, and 87% of patients treated with CTV-margins of 5 mm, 10 mm, and 15–20 mm, respectively. The comparable pattern of failure implies that tumor infiltration was as effectively targeted when patients were irradiated with reduced versus conventional CTV-margins.

Pattern of failure analysis typically examines the spatial relationship between the recurrence volume and the high-dose radiation treatment field (e.g., the 95% isodose line of a radiotherapy plan). By comparing the pattern of failure from conventional radiotherapy plans with those from (theoretical) radiotherapy plans employing a reduced CTV-margin, one can infer the potential impact of CTV-margin reduction. This approach to pattern of failure analysis has long guided efforts to define more optimal CTV-margins for glioblastoma. Yet, this strategy has its limitations, as the observed pattern of failure inherently depends on the initially-chosen CTV-margin and does not provide information on the potential of other CTV-margins. Exploring the distances between the recurrence volume and GTV may better reveal the spectrum of potentially effective CTV-margins. Moreover, recurrence distance analysis may provide a pathway to exploring the concept of personalized CTV-margins. Since the extent of tumor infiltration may vary among patients [10], [11], CTV-margins that are tailored to the individual patient may offer additional value for optimizing target delineation.

In this work, we assessed the distribution of distances between the GTV and recurrence volume in a retrospective cohort of patients with glioblastoma, treated with standard chemoradiation. Furthermore, we performed multiple linear regression modeling to identify variables that were associated with the distance between the GTV and the recurrence volume, and to evaluate the potential for personalized CTV-margins.

## 2 Methods

### 2.1 Patient characteristics and data collection

This retrospective study was approved by the Institutional Review Board of the Erasmus MC, University Medical Center Rotterdam, Rotterdam, The Netherlands (reference number: MEC-2022-0760; date of approval: 03 January 2023). Between January 2012 and December 2022, 302 adult patients with pathologically confirmed newly diagnosed IDH-wildtype glioblastoma (in accordance with the 2021 WHO Classification of Tumors of the Central Nervous System [12]), were scheduled to receive radiotherapy with a total dose of 60 or 40.05 Gray (Gy) at the Department of Radiotherapy of the Erasmus MC Cancer Institute (Rotterdam, The Netherlands). Patients were excluded from this analysis if they did not complete radiation treatment (*n* = 11), they did not receive concurrent chemotherapy (*n* = 6), their radiotherapy treatment plan was unavailable (*n* = 3), progressive disease (PD) was not confirmed (*n* = 57), follow-up MRI was unavailable (*n* = 16), or pathological analysis after re-resection revealed PD was pseudoprogression rather than true tumor recurrence (*n* = 8). For the remaining 201 patients, we collected clinical and imaging data, the radiotherapy target delineations, and the delivered radiotherapy treatment plans. The following parameters were collected for multiple linear regression modeling: age (at the time of surgery), gender, tumor laterality (hemisphere), the presence of contrast-enhancement prior to surgery, extent of resection, O^6^-methylguanine methyltransferase (MGMT) methylation status, GTV volume, the 5th percentile of the pre-radiotherapy apparent diffusion coefficient (ADC) signal intensity distribution within the GTV (ADC_P5), and involvement of the subventricular zone (SVZ) and subgranular zone (SGZ). The GTV typically encompassed the resection cavity plus any residual contrast-enhancing tumor on post-contrast T1-weighted MRI. Deviations from this definition were possible at the discretion of the treating radiation oncologist. The choice of the 5th percentile of the ADC distribution (ADC_P5) was motivated by its robustness to outlier voxels with erroneously low signal intensities. The ADC_P5 is an indicator of diffusion restriction, with lower values associated with higher Ki-67 labeling indices [13]. The SVZ was considered involved when the contrast-enhancing tumor touched the lining of the lateral ventricles on preoperative imaging [14], [15], [16], [17]. For the SGZ, the pre-radiotherapy T1-weighted image was first skull-stripped (HD-BET [18]) and then registered to the MNI152 brain template (*flirt* [19], [20], FSL v6.0.7, Oxford, UK). The resulting transformation matrix was used to register the GTV to MNI152 space. The SGZ was considered involved when the GTV and the SGZ mask from the Hippocampus and Subfields CoBrA atlas [21] overlapped.

### 2.2 Recurrence analysis

**Fig. 1:**
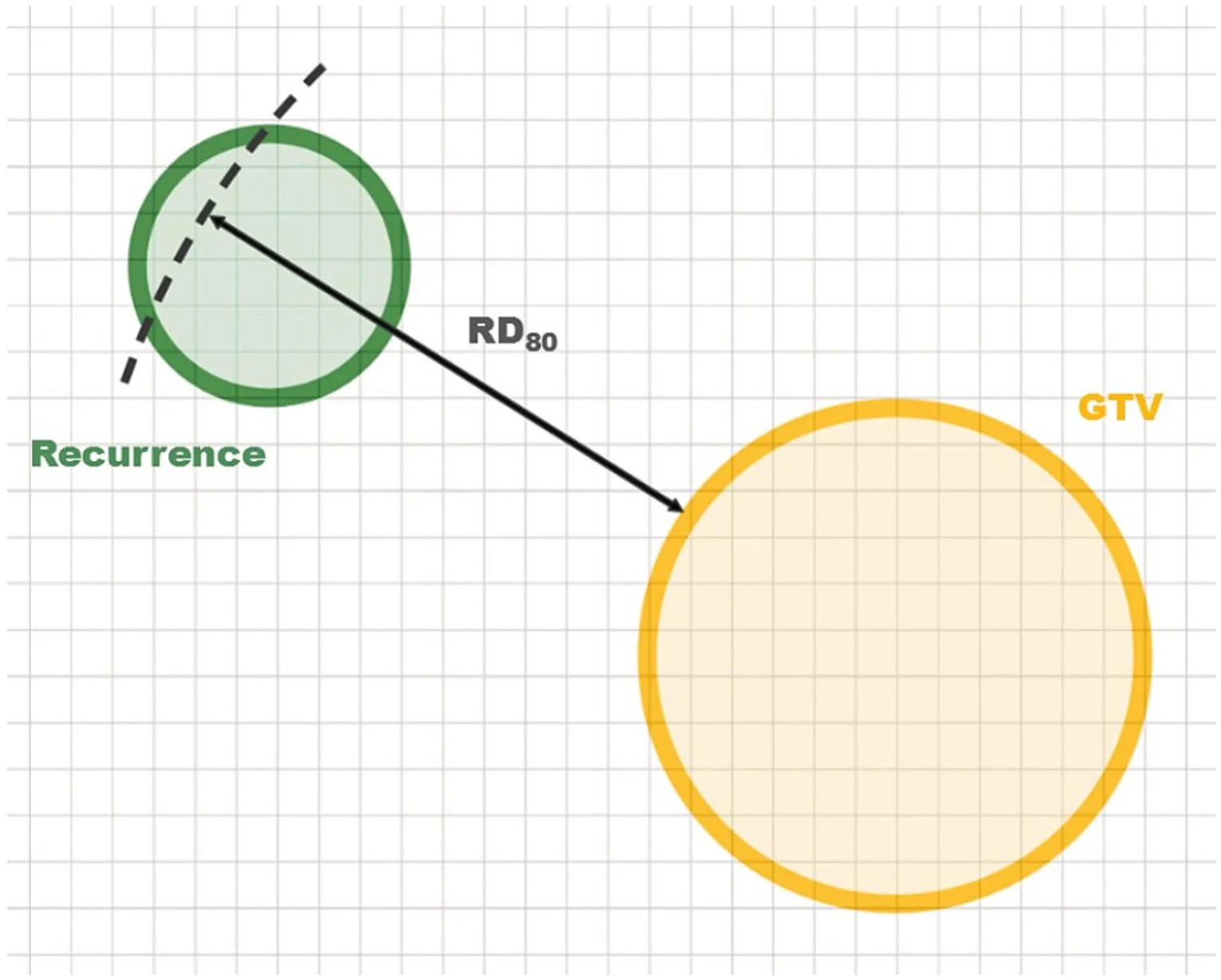
A schematic representation of the recurrence distance calculation. The RD_80_ represents the minimum isotropic expansion from the GTV required to encompass 80% of the recurrence volume. If a margin of RD_80_ was added to the GTV, 80% of the recurrence volume would be located within this margin. Note that we show a simplified 2-dimensional representation in this figure.

The timepoint of recurrence was defined as the moment when the multidisciplinary team or treating physician determined PD. Sequential MRI-scans obtained before and after this timepoint were reviewed in consultation with a neuroradiologist to confirm radiological evidence of progression and reduce the risk of misclassification due to pseudo-progression. The MRI-scan showing first radiological evidence of progression was rigidly registered to the planning CT in MIM Maestro® (v7.1.6), and alignment was visually verified for all cases. Subsequently, the contrast-enhancing recurrence volume was manually delineated in MIM Maestro® by a single observer. The recurrence distance was defined as the minimum isotropic expansion from the GTV required to encompass 80% of the recurrence volume (RD_80_). Thus, an RD_80_ of 15 mm indicates that a 15 mm isotropic margin around the GTV would encompass 80% of the recurrence volume. The choice for an 80% coverage of the recurrence volume was guided by various studies that performed pattern of failure analysis based on the high-dose radiation treatment field. In these studies, recurrences were classified as in-field if ≥80% of the recurrence volume was located within the 95% isodose line [7], [22], [23], [24]. The RD_80_ was calculated in two steps: First, the minimum Euclidean distance to the outer edge of the GTV was calculated for each voxel within the recurrence volume. Thereafter, the 80th percentile of these distances was extracted to quantify the RD_80_. A schematic representation of the RD_80_ is given in Fig. 1*.* In addition to the RD_80_, we performed pattern of failure analysis based on the high-dose radiation treatment field. Recurrences were classified as in-field, marginal, or distant if >80%, 20–80, <20% of the recurrence volume was covered by the 95% isodose line of the clinical radiotherapy plan, respectively.

### 2.3 Regression analysis

Because visual assessment of the GTV volume and the RD_80_ showed right-skewed distributions, both variables were log-transformed using the natural logarithm. For the RD_80_, a small constant of 0.5 mm was added to all distances prior to log-transformation to account for zero values. We performed multiple linear regression analyses to identify relevant variables that were associated with the log-transformed RD_80_. First, all variables presented in Table 1, excluding the CTV-margin and number of adjuvant chemotherapy cycles until PD, were examined using univariable linear regression. Variables with a *p*-value <0.20 were retained for the initial multiple linear regression model. As involvement of neurogenic zones has been found to correlate to distant recurrences and dissemination [14], [15], [16], [17], [25], [26], [27], [28], the variables involvement of the SVZ and the SGZ were included in the initial multivariable model regardless of their statistical significance in the univariable models. We applied a backward elimination procedure, where the variable with the highest p-value was sequentially removed at each step. Backward elimination continued until solely variables with a statistically significant association (two-sided *p* < 0.05) remained. To assess the impact of using an alternative recurrence distance metric, the minimum isotropic expansion from the GTV required to encompass 95% of the recurrence volume (RD_95_) was additionally computed, and the regression analysis with backward elimination was repeated using the RD_95_. Regression analysis was performed using R (v4.3.2).

**Table 1:** Patient and tumor characteristics (*n* = 201).

Characteristics	No. of patients (%)
**Age (years)**	
Mean (range)	58 (31–81)

**Gender**	
Male	134 (66.7%)
Female	67 (33.3%)

**Hemisphere**	
Left	92 (45.8%)
Right	100 (49.8%)
Bilateral	9 (4.5%)

**Contrast-enhancement prior to surgery**	
Present	180 (89.6%)
Absent	21 (10.4%)

**Extent of resection**	
Biopsy	33 (16.4%)
Partial resection	141 (70.1%)
Gross total resection	27 (13.4%)

**MGMT methylation status**	
Unmethylated	74 (36.8%)
Methylated	58 (28.9%)
Heterogeneous	11 (5.5%)
Unknown	58 (28.9%)

**GTV volume (cm**^**3**^**)**	
Median (IQR)	42.9 (22.6–78.1)

**ADC_P5 (10**^**−6**^ **mm**^**2**^**/s)**	
Median (IQR)⁎	648 (371–734)
	
**Involvement of the SVZ**	
SVZ involved	80 (39.8%)
SVZ not involved	121 (60.2%)

**Involvement of the SGZ**	
SGZ involved	89 (44.3%)
SGZ not involved	112 (55.7%)

**CTV-margin**	
15 mm	133 (66.2%)
20 mm	68 (33.8%)

**Radiotherapy fractionation scheme**	
30 × 2 Gy	141 (70.1%)
15 × 2.67 Gy	60 (29.9%)

**Adjuvant chemotherapy cycles**	
Median (IQR)	6 (3–6)

⁎ ADC maps were missing in 5 patients.

### 2.4 Sensitivity analysis

From May 2018 onwards, the standard CTV-margin that was used at the Department of Radiotherapy of the Erasmus MC Cancer Institute was reduced from 20 mm to 15 mm. Hence, a number of patients in this cohort received radiation treatment with a radiotherapy plan that employed a 20 mm CTV-margin instead of 15 mm. Additionally, variations in radiotherapy fractionation scheme and number of completed adjuvant chemotherapy cycles existed in our cohort. These variations may have had an impact on the location of tumor recurrence. Therefore, sensitivity analyses were performed by adding the CTV-margin, radiotherapy fractionation scheme, and number of completed adjuvant chemotherapy cycles prior to PD as covariates to the final RD_80_ regression model. Additionally, the log-transformed time to progression was added as an additional covariate to the final RD_80_ regression model.

## 3 Results

### 3.1 Patient characteristics

In the 201 patients, the median time between the last radiotherapy fraction and PD was 6.8 months (IQR: 4.1–11.8 months). An overview of the patient and tumor characteristics is provided in Table 1*.*

### 3.2 Recurrence analysis

**Fig. 2:**
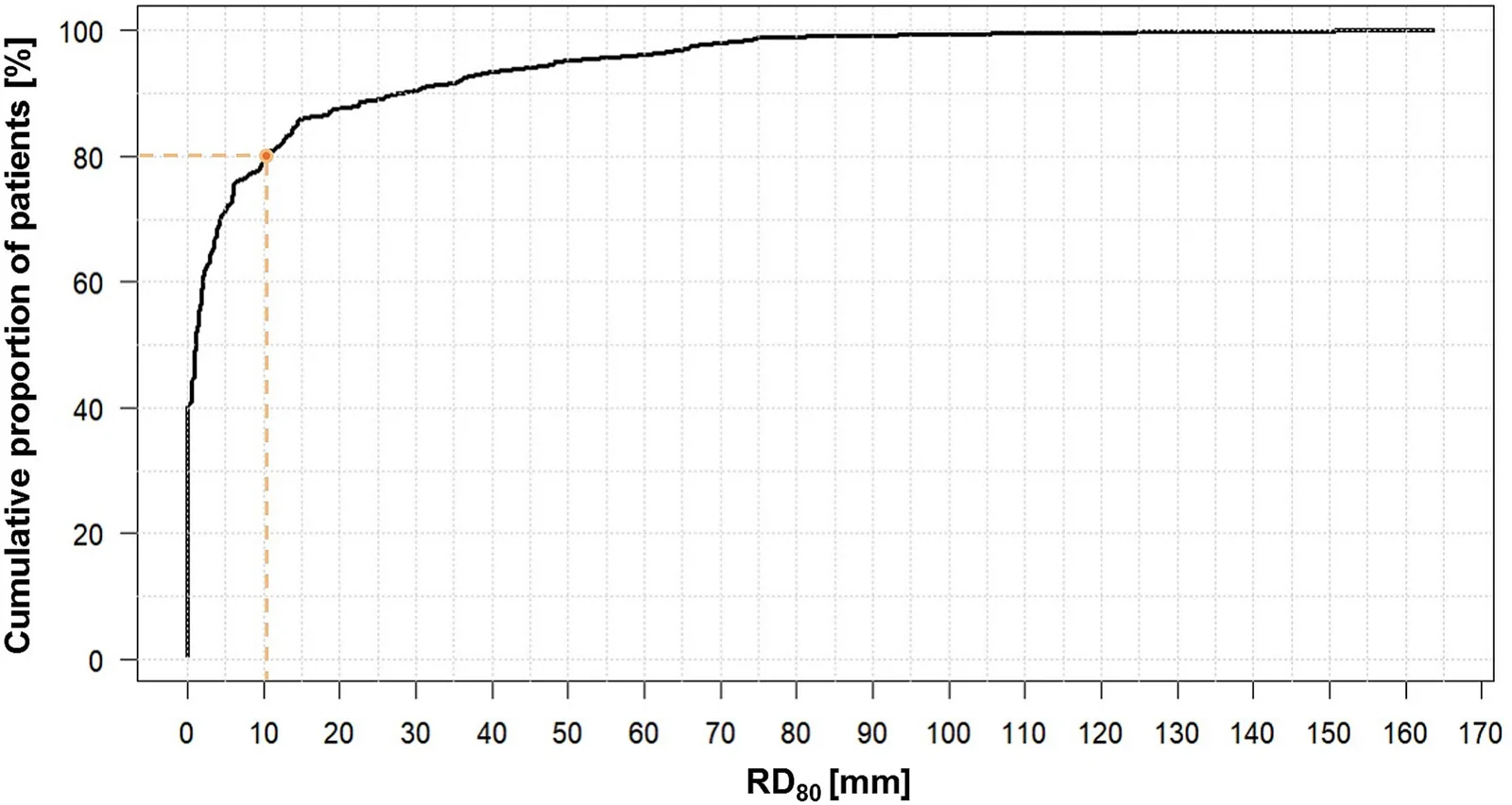
Cumulative distribution of the RD_80_. The vertical axis represents the proportion of patients whose recurrence is located within a specified RD_80_. For instance, the RD_80_ was ≤10.4 mm for 80% of the patients in our cohort (orange dot).

The median recurrence volume was 14.6 cm^3^ (IQR: 1.7–45.0 cm^3^). The median RD_80_ was 1.2 mm (IQR: 0–6.0 mm). Fig. 2 shows the cumulative distribution of the RD_80_ in this cohort. The absolute distribution of the RD_80_ is provided in *Supplementary Fig. S1*. Based on the 95% isodose line of the clinical radiotherapy plan, recurrences were classified as in-field for 175 patients (87.1%), marginal for 6 patients (3.0%), and distant for 20 patients (10.0%).

### 3.3 Regression analysis

From the univariable linear regression *(*Table 2*)*, the variables gender (*p* = 0.08), contrast-enhancement prior to surgery (*p* = 0.18), extent of resection (*p* = 0.05), log-transformed GTV volume (*p* = 3.1e-5), involvement of the SVZ (*p* = 0.09), and radiotherapy fractionation scheme (*p* = 0.06) were identified as potential predictors for the log-transformed RD_80_. The initial multiple linear regression model included the variables gender, contrast-enhancement prior to surgery, extent of resection, log-transformed GTV volume, involvement of the SVZ, involvement of the SGZ, and radiotherapy fractionation scheme. Backward elimination first removed contrast-enhancement prior to surgery (*p* = 0.87) from the initial model, followed by radiotherapy fractionation scheme (*p* = 0.29), extent of resection (*p* = 0.21), and gender (*p* = 0.07) in the subsequent models, resulting in a final model in which the log-transformed GTV volume, involvement of the SVZ, and involvement of the SGZ remained as significant predictors. The (exponentiated) coefficients and *p*-values of the variables in the final multiple linear regression model *(F(3, 197) = 10.3, adjusted R*^*2*^ *= 0.12, overall p-value = 2.5e-6)* are shown in Table 3*.* The initial and intermediate multiple linear regression models are provided in *Supplementary Table S1-S4.* The relationships between the variables retained in the final model and the RD_80_ are provided in *Supplementary Fig. S2,* and the relationships among the variables retained in the final model are provided in *Supplementary Fig. S3.* For the regression analysis with the RD_95_, the log-transformed GTV volume, involvement of the SVZ, and involvement of the SGZ remained as significant predictors after backward elimination. The (exponentiated) coefficients and *p*-values of the final RD_95_ regression model *(F(3, 197) = 11.3, adjusted R*^*2*^ *= 0.13, overall p-value = 6.76e-8)* are provided in *Supplementary Table S5.*

**Table 2:** Univariable linear regression (outcome: log-transformed RD_80_).

Variable		β (coef.)	95% CI	Overall *p*-value
Age		0.01	−0.02–0.03	0.59
Gender⁎	*Male (reference)*	–	–	0.078
	*Female*	−0.42	−0.89–0.05	
Hemisphere	*Left (reference)*	–	–	0.74
	*Right*	−0.07	−0.53–0.39	
	*Bilateral*	0.36	−0.75–1.47	
Contrast-enhancement⁎	*Absent (reference)*	–	–	0.18
	*Present*	0.50	−0.23–1.22	
Extent of resection⁎	*Biopsy (reference)*	–	–	0.053
	*Partial resection*	0.73	0.13–1.34	
	*Gross total resection*	0.76	−0.05–1.57	
MGMT methylation status	*Unmethylated (reference)*	–	–	0.35
	*Methylated*	0.37	−0.15–0.89	
	*Heterogeneous*	0.01	−0.95–0.97	
GTV volume (log-transformed)⁎		−0.52	−0.76 to −0.28	3.1e-5
ADC_P5		−0.00	−0.00–0.00	0.41
Involvement of the SVZ⁎	*Not involved (reference)*	–	–	0.087
	*Involved*	0.40	−0.06–0.85	
Involvement of the SGZ	*Not involved (reference)*	–	–	0.72
	*Involved*	0.08	−0.37–0.53	
Radiotherapy fractionation scheme⁎	*15 × 2.67 Gy (reference)*	–	–	0.055
	*30 × 2 Gy*	0.47	−0.01–0.96	

⁎ Variables with an overall p-value < 0.20.

**Table 3:** Final multiple linear regression model (outcome: log-transformed RD_80_).

Variable		β (coef.)	exp(β)	95% CI	Overall p-value
GTV volume (log-transformed)⁎		−0.70		−0.96 to −0.44	3.9e-7
Involvement of the SVZ⁎	*Not involved (reference)*	–	–	–	0.019
	*Involved*	0.53	1.70	0.09–0.96	
Involvement of the SGZ⁎	*Not involved (reference)*	–	–	–	0.032
	*Involved*	0.51	1.67	0.04–0.98	

⁎ Variables with an overall p-value < 0.05.

### 3.4 Sensitivity analysis

In the sensitivity analyses, the final model was not significantly affected by differences in CTV-margin (β = 0.32, *p* = 0.16) or radiotherapy fractionation scheme (β = 0.32, *p* = 0.18). In contrast, the number of completed adjuvant chemotherapy cycles (β = 0.13, *p* = 0.015) and log-transformed time to progression (β = 0.42, *p* = 0.00075) were independently associated with the RD_80_ when included as additional covariates. When adding both variables simultaneously as covariates to the final RD_80_ regression model, time to progression (β = 0.42, *p* = 0.020) remained significantly associated, whereas number of adjuvant chemotherapy cycles was no longer significant (β = 0.00, *p* = 0.99). In all sensitivity analyses, the independent variables in the model, i.e. log-transformed GTV volume, involvement of the SVZ and involvement of the SGZ, remained significant predictors. The models for the sensitivity analyses are provided in *Supplementary Table S6-S10.* The relationships between the number of adjuvant chemotherapy cycles, time to progression and RD_80_ are provided in *Supplementary Fig. S4.*

## 4 Discussion

In this study, we retrospectively analyzed the distances between the GTV and tumor recurrence in 201 patients with glioblastoma. Recurrence distance analysis demonstrated that 80% of the recurrences occurred within 10 mm from the GTV. Furthermore, GTV volume and involvement of the SVZ and SGZ were significantly associated with the distance between the GTV and tumor recurrence.

Recurrence distance analysis showed that the majority of recurrences in our cohort were located within or in close proximity to the GTV. For 80% of the patients, the RD_80_ was ≤10.4 mm. Notably, the RD_80_ represents a relatively strict metric compared to a pattern of failure classification based on the 95% isodose line. Although we did not generate theoretical radiotherapy plans with a 10 mm CTV-margin, it is reasonable to hypothesize that a pattern of failure analysis using theoretical radiotherapy plans with a 10 mm CTV-margin would have resulted in more than 80% of patients having an in-field recurrence in our cohort. This estimate falls within the range of previously published retrospective data on pattern of failure in patients treated with a 10 mm CTV-margin [7], [8], [9]. However, recurrence distance analysis and pattern of failure analysis based on the high-dose radiation treatment field are not directly interchangeable. Hence, while our work demonstrates that the majority of recurrences are located in close proximity to the GTV, prospective evaluation in clinical trials remains necessary to validate the feasibility of reduced CTV-margins.

In our cohort, smaller GTV volumes were significantly associated with greater distances between the GTV and the recurrence volume. One possible explanation for this association is that smaller tumors exhibit lower hypoxia burden [29]. As a consequence, radiotherapy may have a better local efficacy [30] in patients with smaller GTVs, resulting in a higher likelihood of developing a distant recurrence. Another plausible reason is that the surrounding brain tissue for tumors with larger GTVs becomes more limited, potentially constraining the distance over which tumor infiltration can migrate [31]. It is important to acknowledge that methodological and geometric aspects may also contribute to the observed association. The RD_80_ represents a specific distance from the outer edge of the GTV; hence, it may be influenced by the size and shape of the GTV. The observation that smaller GTV volumes had a higher rate of distant recurrence, is consistent with the results from Langhans et al. [31], who raised the possibility that a CTV-margin depending on GTV size may allow similar (or even improved) tumor control while having less side effects.

Furthermore, contact with a neurogenic zone was a significant predictor of a greater recurrence distance in our cohort, consistent with results from prior studies. Tumors contacting the SVZ have repeatedly been associated with atypical behavior, including a higher likelihood of distant recurrence, multifocal disease, and worse prognosis [14], [15], [16], [17], [25], [26], [27], [32], [33]. Chen et al. [28] further demonstrated that tumors contacting the SVZ or SGZ had a higher incidence of distant recurrence outside the high-dose radiation treatment field. The biological mechanism linking contact with a neurogenic zone and recurrence distance remains unclear. It has been hypothesized that tumor cells in neurogenic zones may co-opt the microenvironment and existing migratory pathways of these regions, potentially enhancing their ability to migrate throughout the brain [26], [34], [35]. Investigating the mechanisms underlying the observed association was beyond the scope of this study and future research aimed at elucidating these mechanisms may provide valuable insights into the biological processes driving glioblastoma recurrence patterns. Interestingly, although involvement of the SGZ was not significantly correlated with the log-transformed RD_80_ in our univariable analysis, it emerged as a significant predictor in the final multiple linear regression model. This change may be explained by confounding. As can be observed in *Supplementary Fig. S3c,* tumors with SGZ involvement tend to have larger GTV volumes. Since our results also indicate that larger GTV volumes are associated with a shorter RD_80_, the association between SGZ involvement and RD_80_ may have been masked by differences in GTV volume. In the multiple linear regression model, this association became apparent after adjusting for differences in GTV volume.

Although our final multiple linear regression model was able to identify significant variables associated with recurrence distance, the model only explained 12% of the variance. This low percentage indicates that it is challenging to reliably predict the recurrence distance with linear regression and the variables that we examined. Nonetheless, the significant variables found in our work may offer a potential direction for personalized CTV-margins.

In our sensitivity analyses, the number of completed adjuvant chemotherapy cycles prior to PD and time to progression were independently associated with the RD_80_ when added separately to the final RD_80_ regression model. The latter finding is in line with previous reports [36], [37]. However, when both variables were added simultaneously, only time to progression remained independently associated, potentially reflecting a positive correlation between time to progression and the number of adjuvant chemotherapy cycles *(see Supplementary Fig. S4e)*. Hence, the association between the number of adjuvant chemotherapy cycles and RD_80_ may partially be reflecting underlying differences in disease course. Further research on the potential impact of adjuvant chemotherapy on recurrence patterns may therefore be of interest. Importantly, the significant predictors in our final model remained unchanged after adjustment for variations in treatment, supporting the robustness of the main findings.

In this study, we introduced the RD_80_ as a novel metric to provide additional quantitative information for pattern of failure analysis in glioblastoma. The 80% coverage threshold for the RD_80_ is not based on a clearly defined biological rationale. Instead, it was chosen to maintain consistency with existing pattern of failure analyses based on the high-dose radiation treatment field, where recurrences are typically classified as in-field when at least 80% of the recurrence volume is located within the 95% isodose line [7], [22], [23], [24]. Lee et al. [38] proposed this cutoff based on an evaluation of the relationship between dose-volume histogram data and the recurrence volume, theorizing that recurrences with ≥80% of their volume located within the 95% isodose line most likely originated within the high-dose radiation treatment field, and subsequently expanded outward. The significant predictors of the final multiple linear regression model remained unchanged when we performed the regression analysis using the RD_95_.

There were several limitations in our study. First, our recurrence distance analysis was designed to explore the potential for reduced CTV-margins. However, this approach does not take into account how recurrence patterns might have changed if a different CTV-margin was actually employed. Nevertheless, our work provides a valuable hypothetical framework for optimizing CTV-margins, laying the groundwork for further prospective exploration. Second, this study only included patients with IDH-wildtype glioblastoma who subsequently developed radiologically confirmed recurrence on follow-up MRI. While this may have introduced selection bias, these criteria were required to allow for recurrence distance analysis in a homogeneous cohort in accordance with the most recent 2021 WHO classification [12]. Third, data on the MGMT methylation status was unknown in 29% of patients, potentially decreasing statistical power to find an effect of MGMT methylation status on the RD_80_ and introducing a selection bias. A proportion of missing data may be attributed to changes in routine molecular diagnostics between 2012 and 2022. Some studies have suggested that MGMT methylation in glioblastoma may be associated with distant recurrences [14], [22], [39]. Data on MGMT methylation status was still available for 143 patients, but did not show a significant association with recurrence distance in our univariable regression. It is also important to acknowledge that we did not include other alterations that may impact glioblastoma recurrence, like EGFR amplification or TERT mutation. Fourth, variability across institutions warrants careful generalizability of our findings. Specifically, our single-institution retrospective design does not account for different contouring philosophies across centers. As a result, our findings may not be directly generalizable to institutions that adopt the cone-down approach outlined in the 2025 ASTRO Clinical Practice Guideline [40]. There were also variations in target delineation within our cohort. While the GTV was typically defined as the resection cavity plus residual contrast-enhancing tumor, deviations from this definition may have occurred in individual cases at the discretion of the treating radiation oncologist (e.g. for non-enhancing tumors). Additionally, 34% of our patients were irradiated with a 20 mm CTV-margin, which could have had an effect on the site of tumor recurrence. Di Perri et al. [41] reported similar pattern of failure and progression-free survival in patients treated with a 20 mm CTV-margin compared to patients treated with a 15 mm CTV-margin, implying the effect may be inconsequential. To obtain further evidence to this effect, we performed a sensitivity analysis confirming that the difference in treated CTV-margin did not significantly impact our results. Fifth, as tumor progression can be accompanied with mass effect and midline shift, registration of the MRI-scan at the time of recurrence to the radiotherapy planning CT can be challenging, and introduce an inaccuracy when calculating the RD_80_. We attempted to minimize these effects by using the MRI-scan with first radiological evidence of recurrence for tumor recurrence delineation. Sixth, we performed multiple linear regression modeling with a backward elimination procedure. This approach may be sensitive to sample-specific variations, potentially leading to model instability. Therefore, the results from our regression analysis should be interpreted with appropriate caution. Finally, we computed the RD_80_ using the Euclidean distance, which does not take anatomical barriers into account. While it may introduce an error in the estimation of the RD_80_, we do not expect a substantial impact on our analysis. From Fig. 2, we can derive that the proportion of patients with an RD_80_ ≤ 15 mm (∼86%) closely matches the proportion of patients with an in-field recurrence (87%) in our cohort, indicating that our approach provides a reasonable estimate.

Our work provides an incentive for prospective clinical trials to assess the effect of reduced CTV-margins on pattern of failure, progression-free and overall survival, and quality of life. While personalized CTV-margins could theoretically benefit patients, our results suggest that the realization of this concept is challenging. Advanced prediction methods, e.g. radiomics [42], may better capture the complex spatial and biological characteristics that drive glioblastoma recurrence patterns. Another approach for more optimal CTV delineation of glioblastoma was introduced by Detsky et al. [43], who demonstrated that MRI-guided adaptive radiotherapy may enable a reduction of the CTV-margin without compromising tumor control. In their study, patients with glioblastoma were treated with a 1.5 Tesla MRI-guided linear accelerator with weekly online adaptation and a 5-mm CTV-margin, resulting in a low rate of marginal failure. Furthermore, future strategies could leverage the preferential infiltration patterns of glioblastoma along white matter tracts and perivascular spaces [44], [45], [46], and shift from isotropic CTV-margins toward patient-specific anisotropic CTVs that more accurately reflect the direction of tumor infiltration. This approach may further benefit from integrating multimodal imaging, like advanced MRI [47] or amino acid PET [48], [49], to enable more accurate target delineation of glioblastoma.

In conclusion, we present the distribution of recurrence distances and significant variables impacting recurrence distance in a large cohort of patients with glioblastoma. Our work demonstrates that the majority of recurrences are located within or in close proximity to the GTV. Prospective evaluation remains necessary to assess the impact of reducing the CTV-margin for patients with glioblastoma.

## CRediT authorship contribution statement

**Patrick L.Y. Tang:** Writing – original draft, Visualization, Software, Project administration, Investigation, Funding acquisition, Formal analysis, Data curation, Conceptualization. **Marion Smits:** Writing – review & editing, Supervision, Funding acquisition, Conceptualization. **Erik D. van Werkhoven:** Writing – review & editing, Validation, Supervision, Methodology, Formal analysis. **Remi A. Nout:** Writing – review & editing, Supervision, Funding acquisition, Conceptualization. **Esther A.H. Warnert:** Writing – review & editing, Supervision, Funding acquisition, Conceptualization. **Alejandra Méndez Romero:** Writing – review & editing, Supervision, Funding acquisition, Conceptualization.

## Funding

This work is supported by the Dutch Research Council (NWO) [grant number 01291481].

## Declaration of competing interest

The authors declare that they have no known competing financial interests or personal relationships that could have appeared to influence the work reported in this paper.
