# Supplementary material for: Recurrence distance analysis for clinical target volume optimization in glioblastoma
Source: Clin Transl Radiat Oncol. 2026 Aug 12;61:101258. doi: 10.1016/j.ctro.2026.101258 (PMC13499103; doi:10.1016/j.ctro.2026.101258)
Supplement: Supplementary file 1 [file mmc1.docx]

*Supplementary materials*

**Recurrence distance analysis for clinical target**

**volume optimization in glioblastoma**

**Supplementary Tables**

Table S1.	………………………………………………………………………………..2

Table S2.	………………………………………………………………………………..3

Table S3.	………………………………………………………………………………..4

Table S4.	………………………………………………………………………………..5

Table S5.	………………………………………………………………………………..6

Table S6.	………………………………………………………………………………..7

Table S7.	………………………………………………………………………………..8

Table S8.	………………………………………………………………………………..9

Table S9.	……………………………………………………………………………....10

Table S10.	……………………………………………………………………………....11

**Supplementary Figures**

Figure S1.	……………………………………………………………………………....12

Figure S2.	……………………………………………………………………………....13

Figure S3.	……………………………………………………………………………....14

Figure S4.	……………………………………………………………………………....15

**Table S1: Initial multiple linear regression model (outcome: log-transformed RD_80_)**

| **Variable** |  | **β (coef.)** | **95% CI** | **Overall**  **p-value** |
| --- | --- | --- | --- | --- |
| Gender | *Male (reference)*  *Female* | --  -0.40 | --  -0.85 – 0.04 | 0.075 |
| Contrast-enhancement | *Absent (reference)*  *Present* | --  -0.061 | --  -0.81 – 0.68 | 0.87 |
| Extent of resection | *Biopsy (reference)*  *Partial resection*  *Gross total resection* | --  0.50  0.37 | --  -0.10 – 1.10  -0.46 – 1.20 | 0.26 |
| GTV volume (Log-transformed) |  | -0.64 | -0.92 – -0.36 | 1.1e-5 |
| Involvement of the SVZ | *Not involved (reference)*  *Involved* | --  0.53 | --  0.08 – 0.99 | 0.022 |
| Involvement of the SGZ | *Not involved (reference)*  *Involved* | --  0.53 | --  0.06 – 1.00 | 0.028 |
| Radiotherapy fractionation scheme | *15 x 2.67 Gy (reference)*  *30 x 2 Gy* | --  0.25 | --  -0.22 – 0.73 | 0.30 |

**Table S2: Intermediate multiple linear regression model 1 (outcome: log-transformed RD_80_)**

| **Variable** |  | **β (coef.)** | **95% CI** | **Overall**  **p-value** |
| --- | --- | --- | --- | --- |
| Gender | *Male (reference)*  *Female* | --  -0.41 | --  -0.85 – 0.04 | 0.071 |
| Extent of resection | *Biopsy (reference)*  *Partial resection*  *Gross total resection* | --  0.49  0.35 | --  -0.09 – 1.07  -0.45 – 1.16 | 0.25 |
| GTV volume (Log-transformed) |  | -0.63 | -0.91 – -0.36 | 9.4e-6 |
| Involvement of the SVZ | *Not involved (reference)*  *Involved* | --  0.53 | --  0.08 – 0.97 | 0.020 |
| Involvement of the SGZ | *Not involved (reference)*  *Involved* | --  0.53 | --  0.06 – 1.00 | 0.027 |
| Radiotherapy fractionation scheme | *15 x 2.67 Gy (reference)*  *30 x 2 Gy* | --  0.26 | --  -0.22 – 0.73 | 0.29 |

**Table S3: Intermediate multiple linear regression model 2 (outcome: log-transformed RD_80_)**

| **Variable** |  | **β (coef.)** | **95% CI** | **Overall**  **p-value** |
| --- | --- | --- | --- | --- |
| Gender | *Male (reference)*  *Female* | --  -0.42 | --  -0.87 – 0.02 | 0.059 |
| Extent of resection | *Biopsy (reference)*  *Partial resection*  *Gross total resection* | --  0.51  0.36 | --  -0.06 – 1.09  -0.45 – 1.16 | 0.21 |
| GTV volume (Log-transformed) |  | -0.66 | -0.93 – -0.40 | 2.3e-6 |
| Involvement of the SVZ | *Not involved (reference)*  *Involved* | --  0.49 | --  0.05 – 0.93 | 0.028 |
| Involvement of the SGZ | *Not involved (reference)*  *Involved* | --  0.53 | --  0.06 – 1.00 | 0.027 |

**Table S4: Intermediate multiple linear regression model 3 (outcome: log-transformed RD_80_)**

| **Variable** |  | **β (coef.)** | **95% CI** | **Overall**  **p-value** |
| --- | --- | --- | --- | --- |
| Gender | *Male (reference)*  *Female* | --  -0.40 | --  -0.85 – 0.04 | 0.072 |
| GTV volume (Log-transformed) |  | -0.70 | -0.96 – -0.44 | 3.7e-7 |
| Involvement of the SVZ | *Not involved (reference)*  *Involved* | --  0.52 | --  0.08 – 0.95 | 0.020 |
| Involvement of the SGZ | *Not involved (reference)*  *Involved* | --  0.53 | --  0.06 – 0.99 | 0.028 |

**Table S5: Final multiple linear regression model (outcome: log-transformed RD_95_)**

| **Variable** |  | **β (coef.)** | **exp(β)** | **95% CI** | **Overall**  **p-value** |
| --- | --- | --- | --- | --- | --- |
| GTV volume (Log-transformed) |  | -0.64 |  | -0.88 – -0.39 | 6.6e-7 |
| Involvement of the SVZ | *Not involved (reference)*  *Involved* | --  0.60 | --  1.82 | --  0.20 – 1.01 | 0.0039 |
| Involvement of the SGZ | *Not involved (reference)*  *Involved* | --  0.66 | --  1.93 | --  0.22 – 1.09 | 0.0034 |

**Table S6: Sensitivity analysis: CTV-margin (outcome: log-transformed RD_80_)**

| **Variable** |  | **β (coef.)** | **95% CI** | **Overall**  **p-value** |
| --- | --- | --- | --- | --- |
| GTV volume (Log-transformed) |  | -0.69 | -0.95 – -0.43 | 5.5e-7 |
| Involvement of the SVZ | *Not involved (reference)*  *Involved* | --  0.54 | --  0.10 – 0.98 | 0.015 |
| Involvement of the SGZ | *Not involved (reference)*  *Involved* | --  0.52 | --  0.06 – 0.99 | 0.029 |
| CTV-margin | *15 mm (reference)*  *20 mm* | --  0.32 | --  -0.12 – 0.76 | 0.16 |

**Table S7: Sensitivity analysis: Radiotherapy fractionation scheme (outcome: log-transformed RD_80_)**

| **Variable** |  | **β (coef.)** | **95% CI** | **Overall**  **p-value** |
| --- | --- | --- | --- | --- |
| GTV volume (Log-transformed) |  | -0.66 | -0.93 – -0.39 | 2.5e-6 |
| Involvement of the SVZ | *Not involved (reference)*  *Involved* | --  0.57 | --  0.13 – 1.01 | 0.012 |
| Involvement of the SGZ | *Not involved (reference)*  *Involved* | --  0.51 | --  0.04 – 0.98 | 0.033 |
| Radiotherapy fractionation scheme | *15 x 2.67 Gy (reference)*  *30 x 2 Gy* | --  0.32 | --  -0.15 – 0.80 | 0.18 |

**Table S8: Sensitivity analysis: Number of completed adjuvant chemotherapy cycles (outcome: log-transformed RD_80_)**

| **Variable** |  | **β (coef.)** | **95% CI** | **Overall**  **p-value** |
| --- | --- | --- | --- | --- |
| GTV volume (Log-transformed) |  | -0.65 | -0.92 – -0.39 | 2.0e-6 |
| Involvement of the SVZ | *Not involved (reference)*  *Involved* | --  0.53 | --  0.09 – 0.96 | 0.017 |
| Involvement of the SGZ | *Not involved (reference)*  *Involved* | --  0.50 | --  0.04 – 0.97 | 0.034 |
| Adjuvant chemotherapy cycles |  | 0.13 | 0.02 – 0.23 | 0.015 |

**Table S9: Sensitivity analysis: Time to progression (outcome: log-transformed RD_80_)**

| **Variable** |  | **β (coef.)** | **95% CI** | **Overall**  **p-value** |
| --- | --- | --- | --- | --- |
| GTV volume (Log-transformed) |  | -0.59 | -0.85 – -0.32 | 2.0e-5 |
| Involvement of the SVZ | *Not involved (reference)*  *Involved* | --  0.51 | --  0.09 – 0.94 | 0.019 |
| Involvement of the SGZ | *Not involved (reference)*  *Involved* | --  0.47 | --  0.01 – 0.93 | 0.045 |
| Time to progression (Log-transformed) |  | 0.42 | 0.18 – 0.66 | 0.00075 |

**Table S10: Sensitivity analysis: Time to progression and number of completed adjuvant chemotherapy cycles (outcome: log-transformed RD_80_)**

| **Variable** |  | **β (coef.)** | **95% CI** | **Overall**  **p-value** |
| --- | --- | --- | --- | --- |
| GTV volume (Log-transformed) |  | -0.59 | -0.85 – -0.32 | 2.2e-5 |
| Involvement of the SVZ | *Not involved (reference)*  *Involved* | --  0.51 | --  0.08 – 0.94 | 0.019 |
| Involvement of the SGZ | *Not involved (reference)*  *Involved* | --  0.47 | --  0.01 – 0.93 | 0.046 |
| Time to progression (Log-transformed) |  | 0.42 | 0.07 – 0.77 | 0.020 |
| Adjuvant chemotherapy cycles |  | 0.00 | -0.15 – 0.15 | 0.99 |

**Figure S1: Distribution of the RD_80_ (a) and log-transformed RD_80_ (b) in absolute count.**

 **(a)**

**(b)**

**Figure S2: Scatterplot and box plots illustrating the association between the (log-transformed) RD_80_ and variables retained in the final multiple linear regression model. GTV volume (a, b) ; involvement of the SVZ (c, d); involvement of the SGZ (e, f).**

**Figure S3: Box plots and table illustrating the association and percentages between the significant variables retained in the final multiple linear regression model. GTV volume – involvement of the SVZ (a, b); GTV volume – involvement of the SGZ (c, d); involvement of the SVZ - involvement of the SGZ (e).**

| **(e)** |  | | **SVZ** | |
| --- | --- | --- | --- | --- |
|  |  |  | **not involved** | **involved** |
|  | **SGZ** | **not involved** | 77 (38.3%) | 35 (17.4%) |
|  |  | **involved** | 44 (21.9%) | 45 (22.4%) |

**Figure S4: Scatter plots illustrating the association between number of completed adjuvant chemotherapy cycles, time to progression and RD_80_. Adjuvant chemotherapy cycles – RD_80_ (a, b); time to progression – RD_80_ (c, d); adjuvant chemotherapy cycles – time to progression (e, f).**
